# Residential variations in hypertension prevalence and trends among adults in Bangladesh

**DOI:** 10.1007/s43999-024-00040-2

**Published:** 2024-03-27

**Authors:** Shahanaj Parvin, Salma Akter, Md. Ismail Hossain, Md. Sabuj Ali, Most Sifat Muntaha Soni

**Affiliations:** 1 Department of Statistics, Jagannath University, Dhaka, Bangladesh; 2 Department of Mathematics and Natural Sciences, BRAC University, Dhaka, 1212 Bangladesh; 3 Department of Statistics, Hajee Mohammad Danesh Science and Technology University, Dinajpur, Bangladesh; 4 Department of Statistics, Islamic University, Kustia, Bangladesh

**Keywords:** High blood pressure, Hypertension, Risk factor, BDHS

## Abstract

**Introduction:**

Hypertension is a widespread and life-threatening condition globally, with its prevalence increasing rapidly, particularly among adults. This study aims to examine the trend in adult hypertension prevalence and associated risk factors in both urban and rural areas of Bangladesh from 2011 to 2018.

**Methods:**

Nationally representative cross-sectional data from the Bangladesh Demographic and Health Survey was used at two time points, 2011 and 2018. In our study, we used a two-step approach for variable selection, combining traditional statistical methods (${\chi }^{2}$ test) with a machine learning algorithm (Boruta algorithm).. This study also employed two different multivariate binary logistic regression models to identify the risk factors that are most closely connected to the presence of hypertension (respectively for urban and rural locations).

**Results:**

According to the study, hypertension has been on the rise in Bangladesh. In 2011, over a third of adults (38.7%) in urban Bangladesh had hypertension, a number that rose by 22.6% in 2017-18. Though rural areas had a lower hypertension prevalence in 2011 (36%), it surged to 64% in 2017-18, surpassing the rate in urban areas. The results of the multivariate analysis showed that age, gender, education, wealth status, area, and survey year had a significant influence on the determinants of hypertension status in connection to place of residence. According to the odds ratio, the prevalence was significantly higher among older respondents, female respondents, wealthy families and higher-educated respondents.

**Conclusion:**

A large proportion of Bangladesh’s adult population suffers from hypertension. A health education program is required to develop appropriate strategies, including appropriate weight control, appropriate physical activity, and healthier eating habits. Health authorities should take initiatives to spread awareness among people, particularly at an older age.

## Introduction

Hypertension, a global prevalent and deadly non-communicable disease, particularly in low and middle-income countries [1, 2]. Globally, around 4 billion people were estimated to have hypertension in 2019, resulting in 10 million deaths [3]. Approximately one-third of adults in low- and middle-income countries suffer from high blood pressure, making hypertension a major contributor to the global burden of disease and death [4]. Based on this, it can be said that hypertension remains one of the leading causes of the global burden of disease and death [5].

The African region has the highest hypertension prevalence among low- and middle-income countries, estimated at 27% by the World Health Organization [6]. A recent population based meta-analysis conducted in Cameroon revealed that one third of the overall adult population suffer from high blood pressure, which leads to stroke and ischemic heart disease [7]. In south Asian context, this rate of prevalence was more than 25% [8].

Bangladesh is one of the low-and middle-income countries that has made significant progress in health areas such as maternal and child health [9]. But this progression is not satisfactory in adult health, especially non-communicable disease. Cardiovascular diseases, diabetes, cancers and chronic respiratory diseases are responsible for two thirds of all deaths in Bangladesh [10]. A recent study reported a 20% increase in adult hypertension prevalence [11]. This highlights hypertension as a growing medical and public health concern in Bangladesh.

The causes of the condition of hypertension diverse and complex. Previous studies based on Demographic and Health Survey (DHS) data have attempted to discover the risk factors for hypertensive status. Age, sex, education, wealth status, working status, caffeinating drink, residence etc. were most important determinants that were associated with hypertension. However, the prevalence of hypertension in different residence types in Bangladesh remains unclear. This study aims to fill this gap by determining the prevalence trend and associated factors of hypertension among the middle-aged and elderly population across all residential locations in Bangladesh. The findings could guide evidence-based interventions to address non-communicable diseases in the country.

## Materials and methods

### Data source

A nationally representative secondary data was used for this study named “Bangladesh Demographic and Health Survey (BDHS)”, which was implemented by the National Institute of Population Research and Training (NIPORT) and funded by the United States Agency for International Development (USAID). This study conducted on BDHS 2011 and BDHS 2017-18 survey data set.

### Sample design

**Fig. 1:**
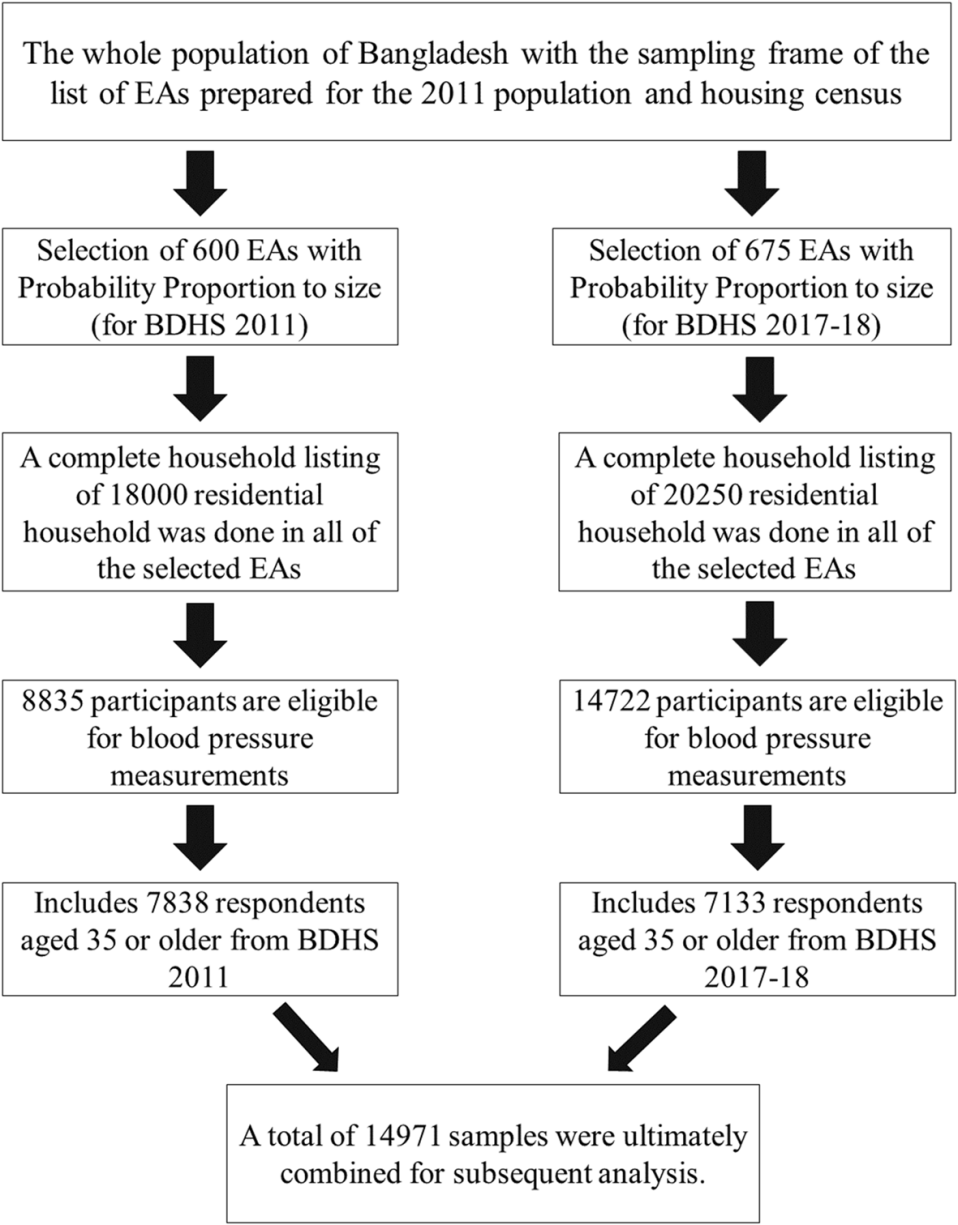
Study population and sample selection procedure for this study

This cross-sectional survey used two stage stratified sampling design, where a list of enumeration areas (EAs) was selected in the first stage, and then households were selected from each enumeration area in the second stage. The survey encompassed 18,000 households, comprising 8,835 respondents for the 2011 survey and 20,250 households with 14,722 respondents for the 2017-18 survey, eligible for blood pressure measurement. Since this study focuses on middle age and older population (i.e., 35+), <35 aged respondents were omitted from this sample. For the further analysis purpose, data were weighted to represent the more accurate structure of Bangladeshi population using weighting factors provided with the Bangladesh Demographic and Health Survey. For hypertension study, after weighing, 7838 (for 2011 survey) and 7133 (for 2017-18 survey) 35+ aged respondents included in this study. After combining the two datasets, there were 14971 adults as a study sample for the analysis. The exclusion and inclusion processes are depicted in Fig. 1.

### Dependent variable

The Bangladesh Demographic and Health Survey includes the collection of blood pressure information from both male and female respondents residing in the households chosen for the survey. According to American Heart Association (AHA) guidelines, the presence of hypertension was counted as if a respondent had systolic blood pressure (SBP) $\ge$ 140 mmHg (millimeters of mercury) or diastolic blood pressure (DBP) $\ge$ 90 mmHg and/or taking antihypertensive medication.

### Independents variables

In choosing the explanatory variables, our primary approach involved conducting a thorough review of pertinent literature related to Bangladesh and assessing the availability of data in the Bangladesh Demographic and Health Survey (BDHS). Multiple socio-demographic and economic variables were included as independent variables such as: respondent age (35-44, 45-54, 55-64, 64+), respondent sex (Male, Female), respondent educational status (No education, Primary education, and Secondary+ education), wealth status (Poor, Middle, and Rich), working status (Yes, No), drink coffee/tea (Yes, No), region (Northern, Eastern, Central, and Southern), and residence (Urban and Rural). Since this study used two survey data set, survey year (2011, and 2017-18) is also an independent variable.

### Statistical analysis

In our study, we employed specific statistical methods to analyze the data and achieve various research objectives. We began with descriptive statistics, examining frequency distributions for all variables. Our variable selection process followed a two-step approach, combining traditional statistical methods with a machine learning algorithm. To explore the relationship between the dependent variable and selected independent variables, we conducted bivariate analysis. In this analysis, we applied the chi-square test, a statistical method to test for independence. Additionally, we incorporated the Boruta algorithm, a machine learning technique that employs a random forest classifier to assess the overall importance of variables. This dual approach, combining traditional statistics and machine learning, enhances the robustness of our variable selection process, providing a more reliable and in-depth understanding of the relationships within the data. In a multivariate setting, this study applied a popular multivariate model called the Binary Logistic with the odds ratio and a 95% confidence interval which was usually used to explain predictor variables impact.

The SPSS (Statistical Package for Social Science) version 25 and R-programming version 4.0.0 was used for data management, analysis.

## Results

Table 1 presents the background characteristics of the respondents participating in the study of hypertension status. The majority of individuals in this section were aged between 35 and 44 years (approximately 36%), with a slight majority being female (50.9%). Participants predominantly belonged to poor and rich households (approximately, 40%, each), while 20.2% were from the middle-class family. Notably, 45% of the total respondent were uneducated, and 9.6% of the total respondents had diabetes problem. Approximately one third of the individuals are from the central region (31%). Most of their (76%) residence is in the rural area. In the 2011 survey, the percentage was larger at 52% compared to 2017-18.

**Table 1:** Percentage distribution of the selected variables for hypertension status

***Variables***	***Frequency (n=14,971)***	***Percentage (%)***
**Respondent age (in years)**		
35-44	5397	36.1
45-54	4112	27.5
55-64	2787	18.6
$\ge$ 65	2675	17.9
**Respondent sex**		
Male	7352	49.1
Female	7619	50.9
**Respondent education**		
None	6774	45.2
Primary education	4335	29.0
Secondary and above	3862	25.8
**Wealth status**		
Poor	5918	39.5
Middle	3022	20.2
Rich	6031	40.3
**Currently working**		
Yes	8355	55.8
No	6617	44.2
**Diabetes**		
Yes	1364	9.6
No	12834	90.4
**Drinking coffee/tea**		
Yes	896	94.0
No	14073	6.0
**Region**		
Northern	3986	26.6
Eastern	3398	22.7
Central	4709	31.5
Southern	2878	19.2
**Residence**		
Urban	3635	24.3
Rural	11337	75.7
**Years**		
2011	7838	52.4
2017-18	7133	47.6

Table 2 illustrates the prevalence of hypertension status and the background characteristics of the selected variables. The ${\chi }^{2}$ test revealed significant associations with hypertension status for all variables $(P< 0.001; P< 0.01; P< 0.05)$. The percentage of respondent with hypertension was notably higher among the older age population ($\ge$ 65 years, approximately 43%), female respondent (34.3%), respondent with secondary and above education (31%), respondent with rich wealth status (approximately 34%), unemployed respondent (34.7%), having diabetes (39%), respondent who drink any coffee/tea (33.6%), respondent who live in southern region in Bangladesh (33.1%), urban residence (33.5%), and for 2017-18 survey year (39.5%).

**Table 2:** Association between selected variables and hypertension status among middle aged and older population in Bangladesh

***Variables***	***Hypertension status***			
	***No (%)***	***Yes (%)***	${{\varvec{\chi}}}^{2}$ ***value***	***p-value***
**Respondent age (in years)**				
35-44	79.00	21.00	490.09	<0.001
45-54	71.60	28.40		
55-64	64.20	35.80		
$\ge$ 65	56.60	43.40		
**Respondent sex**				
Male	74.8	25.2	147.20	<0.001
Female	65.7	34.3		
**Respondent education**				
None	70.1	29.9	5.84	0.04
Primary education	71.4	28.6		
Secondary and above	69.0	31.0		
**Wealth status**				
Poor	73.6	26.4	81.90	<0.001
Middle	71.4	28.6		
Rich	66.2	33.8		
**Currently working**				
Yes	74.1	25.9	138.10	<0.001
No	65.3	34.7		
**Diabetes**				
Yes	60.9	39.1	60.64	<0.001
No	71.1	28.9		
**Drinking coffee/tea**				
Yes	66.4	33.6	6.55	0.01
No	70.4	29.6		
**Region**				
Northern	68.2	31.8	43.11	<0.001
Eastern	73.2	26.8		
Central	71.8	28.2		
Southern	66.9	33.1		
**Residence**				
Urban	66.5	33.5	30.66	<0.001
Rural	71.4	28.6		
**Years**				
2011	79.1	20.9	619.27	<0.001
2017-18	60.5	39.5		

**Fig. 2:**
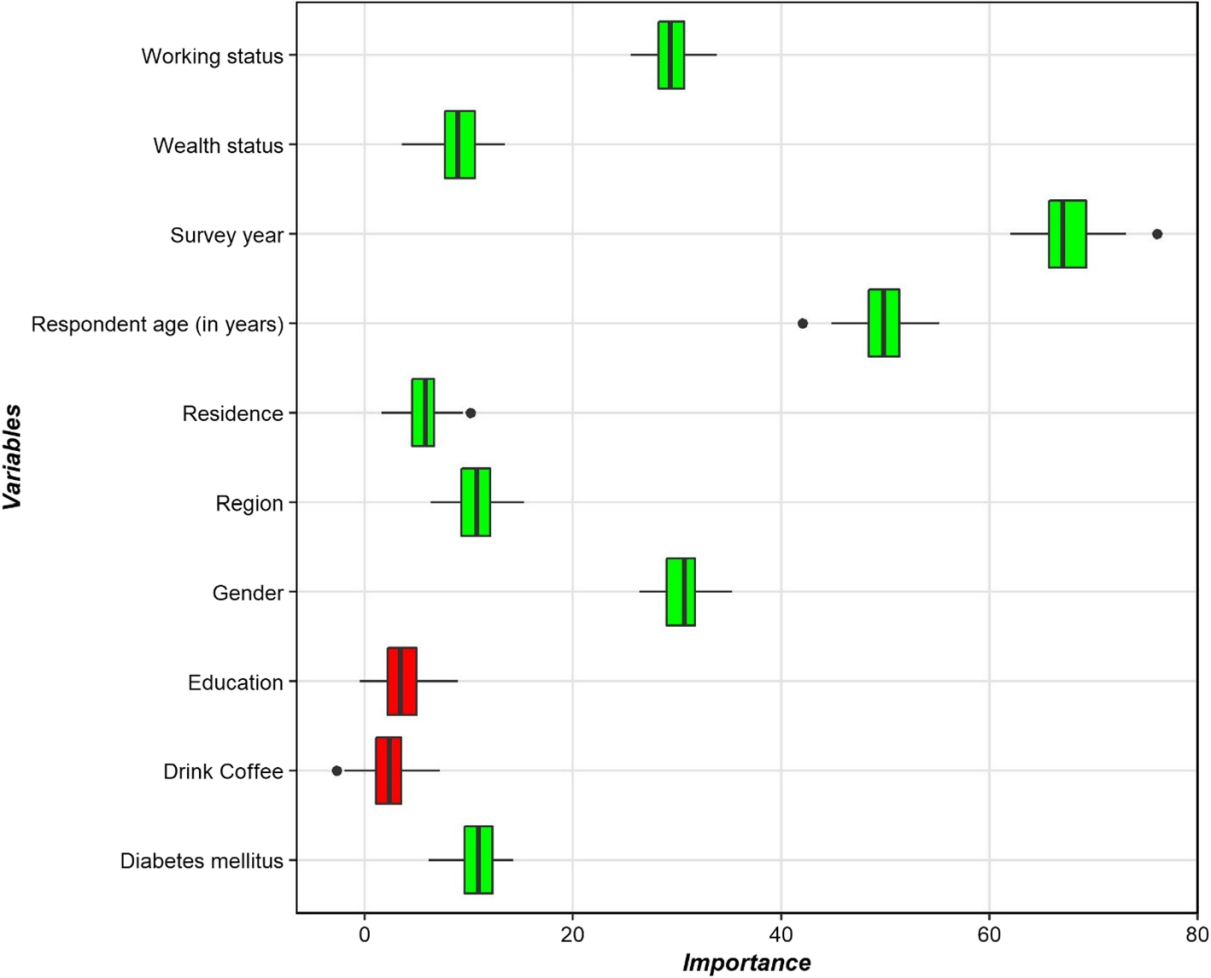
Variable selection using the Boruta algorithm

In Fig. 2, the Boruta algorithm identified eight variables (working status, wealth status, survey year, respondent age, residence, region, gender, diabetes mellitus) as the most crucial risk factors (depicted in the green box plot). Notably, the final analysis of this study excluded unimportant characteristics (shown in the red box plot).

**Fig. 3:**
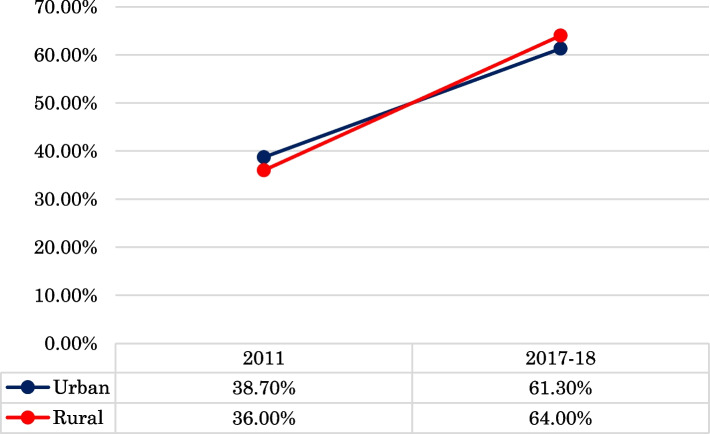
Trends of hypertension prevalence by residential area of Bangladesh

Figure 3 depicts the prevalence trends of hypertension status among adults in both urban and rural Bangladesh. This figure shows a clear increasing trend of hypertension in Bangladesh. In urban Bangladesh, more than one third of adults suffered from hypertension in 2011, which increased by 22.6% in 2017-18. In rural areas, the rate of hypertension was lower than in urban areas in 2011 (approximately 36%). But, in 2017-18, the prevalence rate of hypertension was 64%, which was higher than urban residences in Bangladesh.

Table 3 presents the results of the binary logistic regression model, incorporating the important variables extracted by the Boruta algorithm. It indicates that the risk of hypertension is lower in rural areas of Bangladesh (OR = 0.85) and higher in the year 2017-18 (OR = 2.70). In urban areas, respondents aged 45-54 years, 55-64 years, and >64 years have 1.67 times, 2.38 times, and 2.95 times higher risk of hypertension (respectively) than respondents in the 35-44 years age group. Female respondents, compared to males, have a higher risk of hypertension in both urban (OR = 1.41) and rural (OR = 1.51) residences.

**Table 3:** Binary logistic regression analysis showing the risk of hypertension status among middle and older aged population by background characteristics in urban and rural residence

***Variables***	**Full sample**			**Urban sample**			**Rural sample**		
	***OR***	***95% CI***	***p-value***	***OR***	***95% CI***	***p-value***	***OR***	***95% CI***	***p-value***
**Respondent age (in years)**									
35-44 (ref.)	1			1			1		
45-54	1.62	1.46, 1.79	**<0.001**	1.67	1.39, 2.02	**<0.001**	1.60	1.42, 1.80	**<0.001**
55-64	2.08	1.86, 2.32	**<0.001**	2.38	1.92, 2.96	**<0.001**	1.99	1.76, 2.26	**<0.001**
>64	3.07	2.74, 3.45	**<0.001**	2.95	2.32, 3.75	**<0.001**	3.11	2.73, 3.55	**<0.001**
**Gender**									
Male (ref.)	1			1			1		
Female	1.49	1.35, 1.64	**<0.001**	1.41	1.16, 1.71	**<0.001**	1.51	1.35, 1.69	**<0.001**
**Wealth status**									
Poor (ref.)	1			1			1		
Middle	1.10	0.96, 1.22	0.259	1.22	0.89, 1.69	0.302	1.10	0.98, 1.23	0.301
Rich	1.47	1.34, 1.61	**<0.001**	1.99	1.55, 2.57	**<0.001**	1.36	1.23, 1.52	**<0.001**
**Working status**									
Yes (ref.)	1			1			1		
No	1.25	1.13, 1.39	**<0.001**	1.15	0.94, 1.41	0.203	1.31	1.16, 1.48	**<0.001**
**Diabetes mellitus**									
Yes (ref.)	1			1			1		
No	0.74	0.65, 0.84	**<0.001**	0.74	0.59, 0.90	**0.006**	0.75	0.64, 0.87	**<0.001**
**Region**									
Northern (ref.)	1			1			1		
Eastern	0.65	0.58, 0.72	**<0.001**	0.69	0.54, 0.88	**0.003**	0.63	0.56, 0.72	**<0.001**
Central	0.73	0.66, 0.81	**<0.001**	0.72	0.59, 0.90	**0.005**	0.71	0.63, 0.80	**<0.001**
Southern	0.95	0.85, 1.06	0.324	0.82	0.64, 1.06	0.109	0.99	0.87, 1.12	0.731
**Residence**									
Urban (ref.)	1			-	-	-	-	-	-
Rural	0.85	0.77, 0.93	**0.002**	-	-	-	-	-	-
**Survey year**									
2011 (ref.)	1			1			1		
2017-18	2.70	2.50, 2.93	**<0.001**	2.27	1.95, 2.65	**<0.001**	2.92	2.66, 3.20	**<0.001**

In urban areas, the risk of hypertension is significantly 1.99 times higher for rich households compared to poor households, whereas in rural areas, the respective odds are 36% higher for rich households in Bangladesh. Unemployed respondents have a higher risk of hypertension than employed respondents (OR = 1.15 for urban and OR = 1.31 for rural residences in Bangladesh). Both urban and rural respondents without diabetes are less likely to have hypertension than those with diabetes (OR = 0.74 for urban and OR = 0.75 for rural). For both residences, households from the eastern (OR = 0.69 for urban and OR = 0.63 for rural) and central regions (OR = 0.72 for urban and OR = 0.71 for rural) are significantly less likely to be at risk of hypertension than those from the northern region in Bangladesh.

## Discussion

The findings of the current study indicate and increasing trend in hypertension prevalence in Bangladesh. According to the results, there was a 19% rise in the prevalence of hypertension from year 2011 to year 2017-18. These results align with previous study conducted in Bangladesh [12–14]. High hypertension risk was significantly associated with respondent age, , wealth status, respondent working status, and geographical region in both urban and rural areas of Bangladesh.

This study highlights that older individual are at a higher risk of hypertension compared to middle aged individuals, a consistent finding supported by evidence from several studies [15–18]. Geographical location was also a significant factor, with the eastern and central regions of Bangladesh having lower risk of hypertension than northern region, aligning with similar observations [19].

A noteworthy positive association was found between hypertension and household wealth status. Respondents from middle-class and upper-class families were more likely to have hypertension than those from poor families. This aligns with findings from several previous studies [20–22]. The study reveals that females exhibit a substantially higher prevalence of hypertension than male respondents. The likelihood of female adults developing hypertension increases with age and becomes more significant than in men [23, 24]. These results differ from an Indian study where males had a higher probability of developing hypertension than females [25, 26].

## Strengths and limitations of the study

The study excels in using data that represents the whole country to uncover the factors contributing to differences in hypertension prevalence across regions in Bangladesh. By employing a dual-variable selection approach, we strengthen our process of selecting variables, leading to a more reliable and in-depth understanding of the connections within the data.

However, it's crucial to acknowledge certain limitations. Firstly, due to data constraints, the study couldn't include several significant factors influencing hypertension prevalence in Bangladesh. Secondly, because of the study's cross-sectional design, which examines data at a specific point in time, establishing cause-and-effect relationships wasn't feasible.

## Conclusion

This study attempted to assess the prevalence of hypertension in Bangladesh and attempted to provide comprehensive summary estimates of the prevalence of hypertension along with their trend. According to this study, the prevalence of hypertension remains high and increasing in Bangladesh. Therefore, health programs based on risk factor prevention could help reduce the prevalence of the disease. Initiatives to raise awareness among young people, including special care for the elderly and those at risk of non-communicable diseases. Other effective interventions include maintaining body weight, encouraging physical activity and leading a healthy lifestyle.

## Data Availability

In this study, we used data from Bangladesh Demographic Health Survey (BDHS), 2011 and 2017-18, which is available from https://dhsprogram.com/data/available-datasets.cfm.
